# Mental illness related disparities in diabetes prevalence, quality of care and outcomes: a population-based longitudinal study

**DOI:** 10.1186/1741-7015-9-118

**Published:** 2011-11-01

**Authors:** Qun Mai, C D'Arcy J Holman, Frank M Sanfilippo, Jonathan D Emery, David B Preen

**Affiliations:** 1 School of Population Health, The University of Western Australia, 35 Stirling Highway, Crawley, WA 6009, Australia; 2 School of Primary, Aboriginal and Rural Health Care, The University of Western Australia, 35 Stirling Highway, Crawley, WA 6009, Australia

## Abstract

**Background:**

Health care disparity is a public health challenge. We compared the prevalence of diabetes, quality of care and outcomes between mental health clients (MHCs) and non-MHCs.

**Methods:**

This was a population-based longitudinal study of 139,208 MHCs and 294,180 matched non-MHCs in Western Australia (WA) from 1990 to 2006, using linked data of mental health registry, electoral roll registrations, hospital admissions, emergency department attendances, deaths, and Medicare and pharmaceutical benefits claims. Diabetes was identified from hospital diagnoses, prescriptions and diabetes-specific primary care claims (17,045 MHCs, 26,626 non-MHCs). Both univariate and multivariate analyses adjusted for socio-demographic factors and case mix were performed to compare the outcome measures among MHCs, category of mental disorders and non-MHCs.

**Results:**

The prevalence of diabetes was significantly higher in MHCs than in non-MHCs (crude age-sex-standardised point-prevalence of diabetes on 30 June 2006 in those aged ≥20 years, 9.3% vs 6.1%, respectively, *P *< 0.001; adjusted odds ratio (OR) 1.40, 95% CI 1.36 to 1.43). Receipt of recommended pathology tests (HbA_1c_, microalbuminuria, blood lipids) was suboptimal in both groups, but was lower in MHCs (for all tests combined; adjusted OR 0.81, 95% CI 0.78 to 0.85, at one year; and adjusted rate ratio (RR) 0.86, 95% CI 0.84 to 0.88, during the study period). MHCs also had increased risks of hospitalisation for diabetes complications (adjusted RR 1.20, 95% CI 1.17 to 1.24), diabetes-related mortality (1.43, 1.35 to 1.52) and all-cause mortality (1.47, 1.42 to 1.53). The disparities were most marked for alcohol/drug disorders, schizophrenia, affective disorders, other psychoses and personality disorders.

**Conclusions:**

MHCs warrant special attention for primary and secondary prevention of diabetes, especially at the primary care level.

## Background

Mental illness carries high risks of morbidity and mortality from physical illness [1-3]. Disparities in access to and the quality of physical health care may contribute to poor physical health outcomes in people with mental illness [4]. Access to care is a prerequisite for quality of care, whilst primary care is a foundation for population health, especially for vulnerable groups, including people with mental illness [5]. Our previous study found that Western Australian mental health clients (MHCs), except homeless MHCs (4%), had substantially more visits to general practitioners (GPs) than non-MHCs [6]. This suggests that, in Australia, with its universal health insurance coverage provided by Medicare [7], it appears unlikely that limited access to primary care explains poor physical health outcomes in MHCs. We, therefore, turned our focus to potential disparities in the quality of primary care using diabetes care as an indicator [8], because: (i) diabetes is a major medical condition and a growing epidemic in the general population [9], (ii) the evidence base for diabetes care is strong, and (iii) primary care is the ideal setting for diabetes care.

Previous research has been limited by cross-sectional study design, focus only on one single mental disorder, or an inability to capture care received outside a particular payment system. Few studies have applied rigorous methods to define the non-diabetic population, or are population-based, using linked data, or have examined the association with long-term outcomes.

To address these gaps, we used population-based linked data to answer four specific questions: (i) is diabetes prevalence greater in MHCs; (ii) are diabetes quality of care measures worse in MHCs; (iii) are risks of hospitalisation for diabetes complications, diabetes-related mortality and all-cause mortality higher in MHCs; and (iv) do these associations vary by type of mental disorder?

## Methods

We conducted a population-based longitudinal study for the period 1 January 1990 to 30 June 2006, using the cross-jurisdictional data linkage facility of the Western Australian Data Linkage System (WADLS) [10].

### Data sources

We linked patient-level data from seven datasets in the WADLS, including WA state mental health registry (MHR, 8% of the general population, generally with moderate to severe mental illness), electoral roll registrations (86% of the general population aged ≥18 years), hospital inpatient discharges, emergency department attendances, and death registrations; and Commonwealth Medicare Benefits Scheme claims (MBS, covering GP and pathology services) and Pharmaceutical Benefits Scheme claims (PBS, prescriptions dispensed) (Table 1).

**Table 1:** Summary of study methods

A. Data sources for the study				
***Data sources***	***Period***	***Records***	***Persons***	***Key variables***

1. Medicare benefits (MBS)	1984 to 2006	143,416,764	554,541	GP and pathology services
2. Pharmaceutical benefits (PBS)	1990 to 2006	78,467,656	475,826	Prescriptions
3. Hospital inpatients*	1980 to 2006	4,844,745	520,236	Dates, diagnoses and procedures
4. Mental health registry (MHR)				Dates and diagnoses
- Mental health inpatients*	1966 to 2006	529,935	150,401	
- Mental health outpatients and community	1971 to 2006	9,981,236	190,440	
5. Electoral roll registrations	1988 to 2006	782,161	483,524	Date of registration and updates
6. Death*	1990 to 2006	80,546	80,546	Date of death and cause of death
7. Emergency department*	2001 to 2006	990,506	248,457	Date of attendance and diagnosis

**B. Data sources and codes for identifying diabetes**				

*Hospital inpatient and ED*	*ICD-9 code in any diagnosis field *			

- Diabetes	250			
- Polycystic ovary syndrome	256.4			

*PBS*	*Item*			

- Oral hypoglycaemic agents	1202, 1801, 2178, 2430, 2440, 2449, 2607, 2720, 2939, 2940, 8188, 8189, 8391, 8392, 8450, 8451, 8452, 8533, 8535, 8607, 8687, 8688, 8689, 8690, 8691, 8692, 8693, 8694, 8695, 8696, 8810, 8811, 8838, 8884			
- Insulin	1425, 1426, 1429, 1430, 1431, 1461, 1462, 1531, 1532, 1533, 1534, 1535, 1537, 1591, 1592, 1710, 1711, 1713, 1715, 1716, 1718, 1721, 1722, 1761, 1762, 1763, 2061, 2062, 8006, 8084, 8085, 8212, 8390, 8435, 8571, 8609, 8874, 9039, 9094			

*MBS *	*Item*			

- GP diabetes annual cycle of care	2517, 2518, 2521, 2522, 2525, 2526, 2620, 2622, 2624, 2631, 2633, 2635			
- HbA_1_c or fructosamine	66551, 66554, 66557, 66319, 66322, 73815, 73840			

**C. Category of mental disorders**				

*Category*	*ICD-9 principal code*			

- Alcohol/drug disorders	291, 292, 303 to 305			
- Schizophrenia	295			
- Affective psychoses	296			
- Other psychoses	293, 294, 297 to 299			
- Neurotic disorders	300			
- Personality disorders	301			
- Adjustment reaction	309			
- Depressive disorder	311			
- Other mental disorders	302, 306 to 308, 310, 312 to 319			
- Other than mental or behavioural disorders	not in 290 to 319			

**D. MBS items for identifying pathology tests for routine diabetes monitoring**				

*Test*	*Item*			

Glycated haemoglobin (HbA1c)	66551, 66319			
Microalbuminuria	66361, 66560			
Lipids	66317, 66334, 66335, 66337, 66339, 66341, 66521, 66524, 66527, 66530, 66533, 66536			

**E. ICD-9 codes for identifying diabetes complications and diabetes-related deaths**				

	*ICD-9 principal diagnosis or procedure codes*			

Diabetes/diabetes complications	250			
Circulatory disorders				
- Hypertension	401 to 405			
- Ischemic heart disease	410 to 414			
- Cerebrovascular disease	430 to 438, 362.34, 784.3			
- Heart failure	428, 429.2 to 429.3, 429.9			
- Atherosclerosis	440			
- Peripheral vascular disease	443, 459.8 to 459.9, 444, 447.1			
Visual disorders				
- Glaucoma	365			
- Cataract	366			
- Blindness	369			
Other disorders				
- Nephropathy	580 to 586, V45.1, V56			
Other renal complications				
- Infections of kidney	590			
- Cystitis, urinary tract infection	595, 599.0			
- Proteinuria	791.0			
- Neuropathy/other neurologic symptoms	354, 355, 356.8, 729.2			
- Chronic skin ulcer	707			
- Gangrene	785.4			
- Nontraumatic lower-extremity amputation	84.1, 84.3			
Other complications				
- Candidiasis of vulva and vagina	112.1			
- Chronic osteomyelitis of the foot	730.17			

Abbreviations: ED, emergency department; ICD-9, The International Classification of Diseases, ninth revision* In these datasets, ICD-9 was used for 1980-1987, ICD-9-CM for 1988 to June 1999, and ICD-10-AM from July 1999 onwards. We back mapped ICD-9-CM and ICD-10-AM codes to corresponding ICD-9 codes for this study.

The MHR contained mental health inpatient data from all psychiatric institutions, public and private general hospitals, outpatient data from public mental health clinics, community mental health services and psychiatric residential units. Mental health patients treated only by private psychiatrists or GPs were not included in the MHR.

### Study cohorts

**Figure 1:**
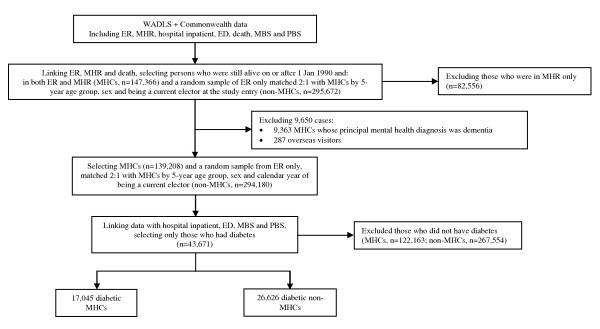
**Selection of study cohorts from the Western Australia Data Linkage System**. Abbreviations: ED, emergency department data; ER, electoral roll registrations; MBS, Medicare Benefits Scheme data; MHCs, mental health clients; MHR, mental health registry; non-MHC, non-mental health clients; PBS, Pharmaceutical Benefits Scheme data; WADLS, the Western Australian Data Linkage System. * Patients who had contact with a community mental health service, but for whom clinicians provided no further information on the number of service contacts. These could be referrals, once-only visits or situations in which health services were not compliant in providing service contact data.

To enhance internal validity of the study, we used the electoral roll as the sampling frame for both MHCs and non-MHCs so that both groups were obtained from the same source population (see Figure 1). MHCs were defined as people on the electoral roll who were also in the MHR (about 80% of MHCs) and still alive from 1 January 1990 onwards. Non-MHCs were a random sample of people who were on the electoral roll, but never recorded in the MHR. They were matched 2:1 with MHCs by five-year age group, sex and current electoral roll registration at study entry. For MHCs, the study entry date was 1 January 1990 for patients first recorded in the MHR before 1 January 1990, or the first date of registration on the MHR for those recorded later. The entry date for non-MHCs was the same as that of their matched MHCs.

Diabetes was identified from hospital discharge diagnoses, prescriptions for glucose lowering medications from the PBS, or diabetes-specific GP services or pathology tests for routine glucose monitoring (HbA_1c _or fructosamine) from the MBS (Table 1). Three exclusions applied: (i) for people identified only by HbA_1c _or fructosamine tests, we included only those with at least three tests in any one year, or at least one test in at least three consecutive years; (ii) people prescribed metformin and concurrently diagnosed with polycystic ovary syndrome; and (iii) people with dementia (due to their high use of residential care). This method of identifying diabetic patients using linked data involved extensive consultation with a clinical advisory group to ensure it accurately reflected clinical management of diabetic patients within WA. For the quality and outcome components of the study, the start of follow-up (T_0_) was the entry date if diabetes was first indicated before then, otherwise it was the date when diabetes was first indicated.

### Variables and measurements

#### Exposure variables

We ascertained the principal mental health diagnosis for each MHC using a previously published method [3], which assigned patients to their most significant mental health diagnosis on an hierarchy of severity (Table 1). People with dementia were excluded after the ascertainment of the principal mental illness. The remaining records were then grouped into one of ten mutually exclusive categories of mental disorders [11].

#### Outcome measures

*Diabetes prevalence*. We calculated the age-sex-standardised point prevalence of diabetes on 30 June 2006 in those aged ≥20 years. The denominators were MHCs and non-MHCs who were still alive on 30 June 2006. The numerators were those in the denominators whose diabetes was recorded on or before 30 June 2006. The 2006 estimated residential population of WA was used as the standard population.

##### Recommended pathology tests

were identified from MBS data indicating a test for HbA_1c_, microalbuminuria or blood lipids (Table 1). Two types of measures were created: (i) cumulative incidence of tests at one year, conditional on at least 365 days of follow-up; and (ii) incidence rate of tests during the entire follow-up period (up to 16.5 years). Two summary measures were also calculated: (i) any test at one year, and (ii) all tests during the entire follow-up period.

##### Other outcome measures

First hospital admissions for a diabetic complication and diabetes-related deaths were identified using Davis' criteria (Table 1) [12]. Diabetes-related deaths included those where diabetes was either the underlying or a contributory cause of death. Follow-up time ended at 30 June 2006 or an earlier date of death or, in the case of first admission for a diabetes complication, on the admission date.

#### Potential confounders

Scores for social disadvantage and residential remoteness were derived from the Index of Relative Socio-Economic Disadvantage (IRSD) [13] and the Accessibility/Remoteness Index of Australia (ARIA) [14] of the Australian Census based on place of residence (address). Social disadvantage scores were grouped into five levels (the lowest 10% of IRSD scores of the WA general population, 10% to < 25%, 25% to < 50%, 50% to < 75% and ≥75%) and remoteness scores were grouped into metropolitan, rural and remote. Diabetic treatment was categorised into: no glucose lowering medications, oral hypoglycaemic agents (OHA) only, and insulin with or without OHA. Age, social disadvantage, residential remoteness and calendar year were measured at T_0 _(or 30 June 2006 for the prevalence study). Physical comorbidities were measured by the Charlson Index [15] based on inpatient data with a five-year look-back period from T_0 _(or 30 June 2006 for the prevalence study). Missing values for each variable were treated as a separate exposure category so that all subjects were included in the multivariate analyses.

### Statistical analyses

Patient characteristics and crude outcomes were compared using bivariate analyses (chi-squared or unpaired tests for categorical variables, two-tailed *t- *or Mann-Whitney tests for continuous variables). We compared MHCs and non-MHCs using univariate and multivariate models for: (i) diabetes prevalence using logistic regression; (ii) receipt of recommended pathology tests using logistic regression (for cumulative incidence) and negative binomial regression (for incidence rates); and (iii) risks of hospitalisation for diabetes complications, diabetes-related mortality and all-cause mortality using Cox regression. Variables adjusted in the multivariate analyses included the five-year age group, sex, Indigenous status, level of social disadvantage, level of residential remoteness, physical comorbidities, calendar year at T_0 _and type of diabetic treatment (the latter two were not included in models for diabetes prevalence). We repeated the above analyses comparing non-MHCs with people in each of the 10 categories of mental disorders. Stata version 10.0 for Windows (StataCorp, College Station, TX, USA) was used for all analyses.

### Ethics approval

The study was approved by the Human Research Ethics Committees of The University of Western Australia, and health departments of the Australian and WA governments.

## Results

### Patient characteristics

The study cohorts comprised 139,208 MHCs and 294,180 non-MHCs. Of these, 17,045 MHCs and 26,626 non-MHCs had diabetes. Characteristics of diabetic patients in MHCs and non-MHCs at T_0 _are shown in Table 2. Relative to diabetic non-MHCs, diabetic MHCs were more likely to be younger, female, Indigenous, socially disadvantaged, living in rural or remote areas, having worse physical health status and being treated with insulin (all *P*-values < 0.001). The distribution of mental disorders among diabetic MHCs is also shown in Table 2.

**Table 2:** Characteristics of diabetic patients in MHCs and non-MHCs at the start of follow up (T_0_)

Patient characteristic*	MHCs (n = 17,045)	Non MHCs (n = 26,626)
Age, years, %		
< 45	20.7%	11.5%
45 to 54	19.7%	14.3%
55 to 64	21.6%	20.7%
65 to 74	18.6%	23.2%
≥75	19.3%	30.3%
Sex, % male	41.9%	43.8%
Indigenous status, %		
Indigenous	11.8%	5.6%
Non-Indigenous	88.1%	91.1%
Missing	0.1%	3.3%
Level of social disadvantage		
Most disadvantaged	15.1%	10.5%
More disadvantaged	22.7%	21.1%
Little disadvantaged	24.5%	23.8%
Less disadvantaged	17.3%	17.8%
Least disadvantaged	19.0%	25.8%
Missing	1.3%	1.0%
Residential remoteness		
Metropolitan	64.8%	70.9%
Rural	25.0%	22.7%
Remote	9.8%	6.3%
Missing	0.4%	0.1%
Charlson comorbidity score, mean (SD)	0.83 (1.62)	0.63 (1.43)
Diabetes recorded before T_0_, %	34.8%	31.6%
Type of treatment at one year, %		
No diabetic medications	53.1%	52.5%
Oral hypoglycemic agents only	39.0%	40.9%
Insulin	7.9%	6.6%
Type of treatment at up to 16 years, %		
No diabetic medications	33.6%	32.6%
Oral hypoglycemic agents only	47.7%	51.0%
Insulin	18.7%	16.4%
Type of mental disorders		
- Alcohol/drug disorders	8.7%	
- Schizophrenia	4.8%	
- Affective psychoses	18.6%	
- Other psychoses	10.7%	
- Neurotic disorders	17.6%	
- Personality disorders	2.2%	
- Adjustment reaction	5.1%	
- Depressive disorder	4.9%	
- Other mental disorders	5.9%	
- Other than mental or behavioural disorders	21.6%	

Abbreviations: MHCs, mental health clients, SD, standard deviation.

*P < 0.001 for all comparisons.

### Diabetes prevalence

**Figure 2:**
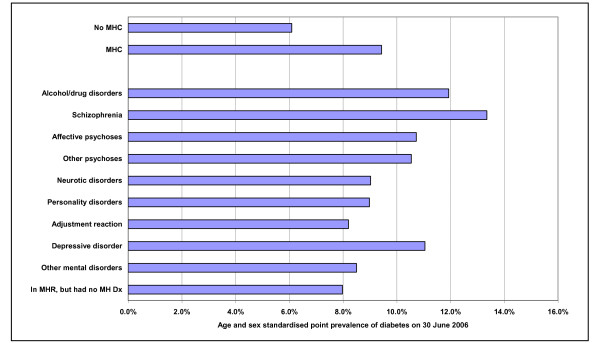
**Point prevalence of diabetic MHCs and non-MHCs on 30 June 2006**. Abbreviations: MHCs, mental health clients; MH Dx, mental health diagnosis; MHR, mental health registry.

**Figure 3:**
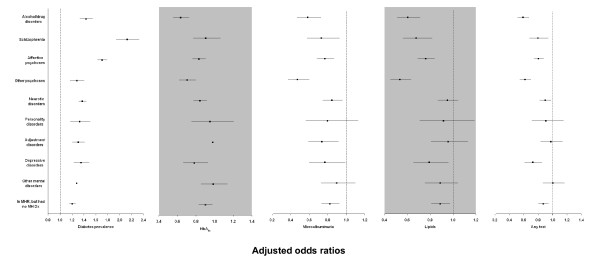
**Multivariate logistic regression for diabetes prevalence and receipt of pathology tests for routine diabetes monitoring at one year of follow-up**. Abbreviations: MH Dx, mental health diagnosis; MHR, mental health registry; non-MHCs, non-mental health clients. Multivariate logistic regression model of diabetes prevalence adjusted for five-year age group; sex; Indigenous status; level of social disadvantage; level of residential remoteness and physical comorbidities. Multivariate logistic regression model of cumulative incidence of pathology test at one year adjusted for five-year age group, sex, Indigenous status, level of social disadvantage, level of residential remoteness, physical comorbidities, calendar year and whether diabetes was identified before T_0 _and type of diabetic treatment. The reference group was non-MHCs.

MHCs with any mental disorders had a higher prevalence of diabetes than non-MHCs (Figures 2 and 3). The overall age-/sex-standardised prevalence at 30 June 2006 for those aged ≥20 was 9.3% vs 6.1%, respectively (*P *< 0.001; Table 3). After adjustment for socio-demographics and case mix, the overall odds ratio (95% CI) was 1.40 (1.36 to 1.43). The prevalence was highest in patients with schizophrenia, affective psychoses and alcohol/drug disorders (Figure 3).

**Table 3:** Disparities in diabetes prevalence, quality of care and outcomes in MHCs and non-MHCs

Measure	MHCs*	Non-MHCs	Unadjusted OR/RR (95% CI)	Adjusted OR/RR (95% CI)†
Diabetes prevalence‡, %	9.3%	6.1%	1.41 (1.37 to 1.44)	1.40 (1.36 to 1.43)
Receipt of recommended test at one year§, %				
- HbA_1c _test	37.2%	42.9%	0.79 (0.76 to 0.82)	0.84 (0.80 to 0.88)
- Microalbuminuria test	12.6%	15.4%	0.79 (0.74 to 0.84)	0.76 (0.71 to 0.82)
- Blood lipid test	24.3%	29.5%	0.77 (0.73 to 0.80)	0.81 (0.77 to 0.85)
- Any test	46.4%	53.2%	0.76 (0.73 to 0.80)	0.81 (0.78 to 0.85)
Receipt of recommended test during the entire follow-up period, rate per person per year				
- HbA_1c _test	0.62	0.68	0.89 (0.86 to 0.91)	0.88 (0.86 to 0.90)
- Microalbuminuria test	0.24	0.27	0.86 (0.83 to 0.89)	0.82 (0.80 to 0.85)
- Blood lipid test	0.40	0.44	0.90 (0.88 to 0.93)	0.90 (0.88 to 0.93)
- All tests	1.25	1.38	0.88 (0.86 to 0.90)	0.86 (0.84 to 0.88)
First hospitalisation for diabetes complications, %	45.2%	45.0%	1.08 (1.05 to 1.12)	1.20 (1.17 to 1.24)
Diabetes related mortality, %	11.8%	11.1%	1.08 (1.02 to 1.15)	1.43 (1.35 to 1.52)
All cause mortality, %	30.4%	27.1%	1.14 (1.10 to 1.19)	1.47 (1.42 to 1.53)
Entire follow-up time, y, mean (SD)	10.5 (5.9)	10.8 (5.6)		

*P < 0.001 for each unadjusted comparison of diabetes measures in MHCs and non-MHCs, except first hospitalisation for diabetes complications (*P *= 0.701) and diabetes related mortality (*P *= 0.020).

† Confounding variables adjusted in the multivariate analyses included the five-year age group, sex, Indigenous status, level of social disadvantage, level of residential remoteness, physical comorbidities, calendar year and whether diabetes was identified before T_0 _and type of diabetic treatment (the latter two variables were excluded from the multivariate analyses for diabetes prevalence).

‡ Age-sex-standardised point prevalence on 30 June 2006.

§ Analyses restricted to subset of patients with at least one year of follow-up (14,836 MHCs and 23,974 non-MHCs).

Abbreviations: 95% CI, 95% confidence interval; MHC, mental health client; OR, odds ratio; RR, rate ratio; SD, standard deviation.

### Receipt of recommended pathology tests

Table 3 shows disparities between diabetic MHCs and non-MHCs in their likelihood of receiving recommended pathology tests for diabetes monitoring at one year and during the entire follow-up period. Unadjusted and adjusted results showed that diabetic MHCs were significantly less likely to receive the pathology tests at one year and during the entire follow-up period. The pattern of results was similar across individual tests (Table 3 and Figure 3). The likelihood of receiving recommended pathology tests were lowest in those with alcohol/drug disorders, other psychoses, depressive disorders and schizophrenia (Figure 3).

### Risks of first hospitalisation for complications of diabetes, diabetes-related mortality and all-cause mortality

**Figure 4:**
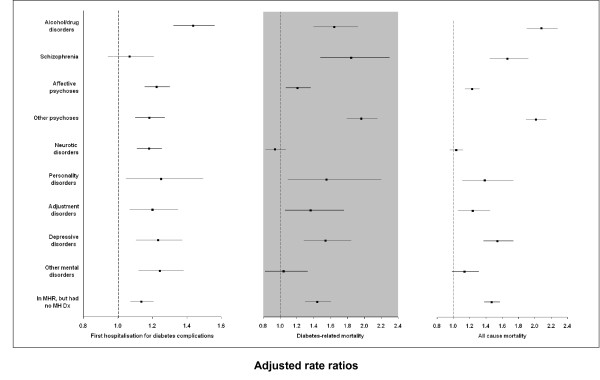
**Multivariate regression for first hospitalisation for diabetes complications, diabetes-related mortality and all-cause mortality**. Abbreviations: MH Dx, mental health diagnosis; MHR, mental health registry; non-MHCs, non-mental health clients. Multivariate models adjusted for five-year age group, sex, Indigenous status, level of social disadvantage, level of residential remoteness, physical comorbidities, calendar year, whether diabetes was identified before T_0 _and type of diabetes treatment. The reference group was non-MHCs.

Diabetic MHCs were at increased risk of hospitalisation for a diabetic complication, or of diabetes-related mortality and all-cause mortality than diabetic non-MHCs in both unadjusted and adjusted analyses (Table 3). Alcohol/drug disorders and personality disorders had the highest risk of hospitalisation for diabetes complications; other psychoses, schizophrenia, alcohol/drug disorders and personality disorders for diabetes-related mortality; and alcohol/drug disorders, other psychoses and schizophrenia for all-cause mortality (Figure 4).

## Discussion

We adopted a population-based approach to examine mental illness-related disparities in disease prevalence, quality of care and outcomes for a major medical condition - diabetes. The results showed that MHCs had higher diabetes prevalence, fewer recommended pathology tests for ongoing diabetes monitoring, and higher risks of hospitalisation for diabetes complications, diabetes-related mortality and all-cause mortality, compared with non-MHCs. Disparities were most marked in MHCs with alcohol/drug disorders, schizophrenia, affective disorders, other psychoses and personality disorders.

The strengths of our study compared with previous published studies are the: (i) use of population-based linked data with over 400,000 people in the study populations, (ii) inclusion of a wide spectrum of mental disorders in the analysis, (iii) use of a comparison group of people with no mental illness, (iv) rigorous identification of diabetes within the study populations, and (v) long-term follow-up (up to 16.5 years).

The limitations of our study included, first, the lack of data for private psychiatrists and GPs treating mental disorders. This limits the extrapolation of our findings for people with mental illness in Australia. Patients in the MHR account for about 40% of people with mental illness (8% of the estimated 20% of Australian adults who have clinically diagnosable mental illness), generally with moderate to severe mental illness. Their physical disease burden and physical health care disparities were probably greater than the remainder of people with mental illness. Nevertheless, using the MHR to identify mental health group as did our previous study [6] ensures the continuity and integrity of our investigations and findings.

Second, while the domain restriction to the WA electoral roll enhanced the internal validity, it reduced the external validity. It is likely that MHCs who were not on the electoral roll have different socio-demographic profiles to MHCs who are on the electoral roll. They may represent a group who are younger than 18 years, have severe mental illness, are homeless, Indigenous, overseas visitors or new migrants [6]. The disparities in this group may be greater. Using Indigenous people as an example, they are known to have high rates of both diabetes and mental illness. They are more likely over-represented in MHCs who were not on the electoral roll and may experience greater barriers to receiving good quality of care. Also the MHR captured only about 40% of patients with mental illness, thus our non-MHC group almost certainly included some people with mental illness. This may result in underestimation of the true difference between MHCs and non-MHCs.

Third, MBS and PBS data do not contain diagnostic information and thus diabetes cases identified from these data are implied rather than definite. However, importantly, we used the same definition to identify diabetes in both groups as the focus of the study was on differences rather than absolute measures. Nevertheless, our diabetes prevalence measures for schizophrenia and affective disorders were consistent with other studies (10 to 15%) [16,17]. Also, regular examinations of eyes and feet, and weight and blood pressure monitoring are all part of a comprehensive ongoing monitoring recommended by the Australian clinical practice guidelines for people with diabetes to prevent the development and progression of macrovascular and microvascular complications [18]. However, MBS does not have enough good data to be able to evaluate these. Fourth, we had no information on lifestyle risk factors or detailed clinical information so the effects of these factors could not be adjusted in the analyses. We also had no information on the clinical decision-making processes leading to pathology testing or hospitalisation, so we can only speculate on the relative roles of patient and doctor in driving or inhibiting the recommended tests or hospitalisation. Last, the ascertainment of diabetes-related death from death certificates may have improved over time, but this would apply equally to both MHCs and non-MHCs leading to negligible change in the relative differences reported in this study.

Previous research on the association between mental illness and diabetes prevalence has exclusively focused on a single mental illness (schizophrenia or affective disorders) and almost all investigations have used the general population as their comparison group [16,17] rather than a non-mental health comparison group we used. Our study showed that all evaluated mental disorders were associated with a higher prevalence of diabetes not just people with severe mental illness. Diabetic MHCs received fewer recommended pathology tests than non-MHCs, after adjustment for socio-demographics and case mix, which is consistent with other studies [19,20], except that we also assessed the long-term disparities (up to 16.5 years). This indicates that the quality of diabetes care may be poorer in MHCs as the differences in the intensity of testing for diabetes monitoring cannot be explained by the differences in socio-demographics, case mix or access to primary care. Our finding that diabetic MHCs had a higher rate of diabetes-related hospitalisations than diabetic non-MHCs suggests that diabetic MHCs may have more diabetic complications than diabetic non-MHCs, possibly due to their lower use of monitoring tests [20]. Another possible reason may be because diabetic MHCs have a lower threshold for diabetes-related admissions than diabetic non-MHCs. Among MHCs, schizophrenia had the lowest relative risk of hospitalisation for diabetes complications, possibly because of their higher diabetes-related mortality rate. In fact, schizophrenia had the highest rate of fatal first complication (adjusted rate ratio 2.81, 1.84 to 4.29).

Possible reasons for MHC-related disparities include factors related to the: (i) patient's cognitive impairment or poor communication skills [21], (ii) provider's bias against 'difficult' patients [22,23], (iii) time constraints of competing conditions [24], and (iv) fragmented health system [25]. The interpersonal aspects of the patient-provider relationship may contribute to more pronounced disparities in quality of care in patients with alcohol/drug disorders and personality disorders [19,26], particularly those with alcohol/drug disorders who are unlikely to receive any preventive care [27].

Our study contributes to emerging evidence which shows that mental illness-related disparities in physical disease burden and physical health care are real and substantial, and present fundamental public health and ethical challenges. While access to care is a prerequisite for good quality care, increasing access may not overcome barriers for good quality care in MHCs. While it is important to promote early detection, diagnosis and treatment of mental illness in primary care settings, attention to physical health conditions in people with existing mental illness is also critical. Given the established overall high rate of GP visits in MHCs [6], there is an opportunity for quality improvement and savings in life-years and costs. Potential interventions to improve physical health care should focus on approaches that highlight both mental health issues and physical care requirements in the consultation. Incentives are required that promote preventive care in the routine management of diabetic patients with comorbid mental illness, particularly those with severe mental illness and behavioural disorders and those with multiple risk factors. Mental and physical health care services need to integrate their efforts to provide a holistic, patient-centred approach to improving health outcomes and quality of life in patients with mental illness and comorbid physical diseases.

## Conclusions

People with mental illness warrant special attention for primary and secondary prevention of diabetes, especially at the primary care level.

## Abbreviations

ARIA: Accessibility/Remoteness Index of Australia; CI: confidence interval; GP: general practitioner; ICD-9-CM: The International Classification of Diseases, 9^th ^revision, Clinical Modification; IRSD: the Index of Relative Socio-Economic Disadvantage; MBS: Medicare Benefits Scheme; MHCs: mental health clients; MHR: mental health registry; OHA: oral hypoglycaemic agents; OR: odds ratio; PBS: Pharmaceutical Benefits Scheme; RR: rate ratio; T_0_: the start of follow up; WA: Western Australia; WADLS: Western Australian Data Linkage System

## Competing interests

The authors declare that they have no competing interests.

## Authors' contributions

QM and CDJH participated in the conception and design of the overall study, and the formulation of the analysis plan. QM researched data and wrote the manuscript. CDJH and FMS reviewed and edited the manuscript and contributed to the discussion. JDE critically revised the manuscript for important intellectual content. DP advised on the method for identifying diabetes using linked data, and reviewed and edited the manuscript. All authors read and approved the final manuscript.

## Authors' information

Qun Mai, MBBS, MPH, Doctoral Scholar, School of Population Health, The University of Western Australia

C D'Arcy J Holman, MPH, PhD, FAFPHM, Chair in Public Health, School of Population Health, The University of Western Australia

Frank M Sanfilippo, BPharm, PGradDipPharm, PhD, Research Assistant Professor, School of Population Health, The University of Western Australia

Jonathan D Emery, MBBCh, DPhil, FRACGP, Chair in General Practice, School of Primary, Aboriginal and Rural Health Care, The University of Western Australia

David Preen, BSc(Hons), PhD, Associate Professor, Director, Centre for Health Services Research School of Population Health, The University of Western Australia

## Pre-publication history

The pre-publication history for this paper can be accessed here:

http://www.biomedcentral.com/1741-7015/9/118/prepub
